# Dietary patterns and risk of non-alcoholic fatty liver disease

**DOI:** 10.1186/s12876-021-01612-z

**Published:** 2021-01-28

**Authors:** Ammar Salehi-sahlabadi, Samaneh Sadat, Sara Beigrezaei, Makan Pourmasomi, Awat Feizi, Reza Ghiasvand, Amir Hadi, Cain C. T. Clark, Maryam Miraghajani

**Affiliations:** 1 Student Research Committee, Department of Clinical Nutrition and Dietetics, Faculty of Nutrition and Food Technology, Shahid Beheshti University of Medical Sciences, Tehran, Iran; 2 Research Committee and Department of Nutrition, School of Nutrition and Food Science, Isfahan University of Medical Sciences, Isfahan, Iran; 3 Department of Nutrition, School of Public Health, Shahid Sadoughi University of Medical Sciences, Yazd, Iran; 4 Nutrition and Food Security Research Center, Shahid Sadoughi University of Medical Sciences, Yazd, Iran; 5 Gastrointestinal and Liver Diseases Research Center, Guilan University of Medical Sciences, PO Box: 73461-81746, Rasht, Iran; 6 Department of Biostatistics and Epidemiology, School of Public Health, Isfahan University of Medical Sciences, Isfahan, Iran; 7 Department of Community Nutrition, School of Nutrition and Food Science, Isfahan University of Medical Sciences, PO Box: 8174673461, Isfahan, Iran; 8 Faculty Research Centre for Sport, Exercise and Life Sciences, Coventry University, Coventry, UK; 9 Cancer Research Center, Shahid Beheshti University of Medical Sciences, Tehran, Iran; 10 Early Life Research Unit, Academic Division of Child Health, Obstetrics and Gynaecology, and Nottingham Digestive Disease Centre and Biomedical Research Centre, The School of Medicine, University of Nottingham, Nottingham, UK

**Keywords:** Dietary patterns, Nonalcoholic fatty liver disease, NAFLD, Factor analysis, Case–control study

## Abstract

**Background:**

Nutrition is a modifiable risk factor that plays an important role in the prevention or delaying of the onset of non-alcoholic fatty liver disease (NAFLD). Previous studies have focused on NAFLD and individual nutrients, which does not take into account combinations of food that are consumed. Therefore, we aimed to investigate the relationship between major dietary patterns and NAFLD.

**Methods:**

This case–control study was conducted on 225 newly diagnosed NAFLD patients and 450 healthy controls. Usual dietary intake over the preceding year was assessed using a validated 168-item semi-quantitative food frequency questionnaire. Major dietary patterns were determined by exploratory factor analysis.

**Results:**

Three dietary patterns, including "western dietary pattern", "healthy dietary pattern", and "traditional dietary pattern" were identified. Subjects in the highest tertile of healthy dietary pattern scores had a lower odds ratio for NAFLD than those in the lowest tertile. Compared with those in the lowest tertile, people in the highest tertile of “western dietary pattern” scores had greater odds for NAFLD. After adjusting for potential confounding factors, “western dietary pattern” had a positive significant effect on NAFLD occurrence. In contrast, “healthy dietary pattern” was associated with a decreased risk of NAFLD. Furthermore, Higher consumption of the “traditional dietary pattern” was significantly associated with NAFLD, albeit in the crude model only.

**Conclusion:**

This study indicated that healthy and western dietary patterns may be associated with the risk of NAFLD. The results can be used for developing interventions in order to promote healthy eating for the prevention of NAFLD.

## Background

Non-alcoholic fatty liver disease (NAFLD) is defined as the presence of fat accumulation in the liver parenchyma in patients without significant alcohol consumption [1]. NAFLD elicits a wide range of hepatic changes and is considered as a chronic liver disease [2]. NAFLD is considered responsible for most cryptogenic chronic liver diseases, and because of its possible progression to non-alcoholic steatohepatitis (NASH), liver cirrhosis and hepatocellular carcinoma [3, 4], it is vitally important that its identification and treatment is focused upon.

NAFLD is an important health issue in both developed and developing countries [5]. Prevalence of NAFLD in the Mediterranean region, Europe, Japan and Singapore is 36.8%, 20–40%, 9–30% and 5% respectively [6, 7]; whilst the prevalence of NAFLD in Iran has been found to be 2.9–7.1% in the general population [8].

NAFLD is strongly associated with central adiposity, type 2 diabetes mellitus (T2DM), insulin resistance, hypertension and metabolic syndrome [9]; whilst some empirical data demonstrates that NAFLD is associated with an increased risk of morbidity and mortality in cardiovascular disease [10, 11]. Obesity is associated with the presence of NAFLD; however, increased central adiposity is asserted a more important indicator of NAFLD than total body fat [12]. Accumulating evidence have indicated that high-calorie diets, especially those rich in saturated and trans fatty acids, and cholesterol, as well as fructose-rich diets, increase central adiposity, visceral fat and the incidence of NAFLD [13]. In addition, certain dietary patterns, such as a western dietary pattern, has been associated with NAFLD, independent of gender, family income and physical activity level [14]. Moreover, it has been shown that dietary pattern is one of the most important factors in preventing and treating NAFLD [15]. One previous cross-sectional study of adults aged 24 to 70 years (n = 375) found an increased risk of NAFLD in those with a higher intake of soft drinks, while a higher intake of omega-3-rich foods reduced the risk [16]. In another study, a high intake of take-out foods, red meat, full-fat dairy products and soft drinks, was associated with higher risk of NAFLD [14]. On the other hand, diet can modify the intestinal microbiota which is considered as and “invisible organ” in human body and can play an important role in normal metabolism and immuno-modulation [17]. The impact of gut microbiota on NAFLD has been suggested by previous studies, it can be a viable target for NAFLD treatment [18].

Given that promising association between foods and nutrients, and risk of NAFLD which have been reported by many studies [19], investigations on dietary patterns and NAFLD are very limited and most studies have focused on the role of single nutrients or foods. Dietary pattern analysis has been used to consider total food intake and the potentially synergistic effects of foods and nutrients which conceivably better reflects the complexity of the diet. Therefore, in this study, we sought to examine the association of the major dietary patterns and the risk of NAFLD among Iranian adults.

## Methods

### Study design and population

This study was a retrospective, age and gender matched, case–control study, which was conducted in the Metabolic Liver Disease Research Center affiliated to Isfahan University of Medical Sciences. Overall, 225 newly diagnosed NAFLD patients and 450 controls were recruited in this study. Individuals who were diagnosed with NAFLD by an expert physician based on laboratory tests and the liver sonography (presence of steatosis) were included in the case group. Healthy individuals based on laboratory tests (alanine aminotransferase [ALT] < 31 UI/L and 41 UI/L and aspartate aminotransferase [AST] < 31 UI/L and 37 UI/L in women and men, respectively) and the liver sonography (not suffering from any stages of hepatic steatosis) were considered as control group. Case and control were matched regarding age and gender.

Individuals with special dietary or physical activity regimens (due to a particular disease or weight loss or professional exercise), history of diseases such as renal and hepatic (Wilson's disease, autoimmune liver disease, hemochromatosis, virus infection, and alcoholic fatty liver), cardiovascular disease, malignancy, thyroid disorder, and autoimmune diseases were excluded from study. Participants who did not complete more than 35 items of the food frequency questionnaire and those who reported total daily energy intakes ≤ 800 or ≥ 4500 kcal/d were also excluded. All participants provided written informed consent prior to the study enrollment.

### Dietary assessment

Dietary intake was assessed using a validated 168-item semi-quantitative food frequency questionnaire (FFQ) [20]. The FFQ included a list of typical Iranian foods with standard serving sizes. Consumption frequencies of each food item were segmented into nine categories. Participants were asked to report their average dietary intake during the previous year by choosing one of the following choices: never or less than once a month, 3–4 times per month, once a week, 2–4 times per week, 5–6 times per week, once daily, 2–3 times per day, 4–5 times per day, and 6 or more times a day. Portion sizes of each food item were converted into grams by using standard Iranian household measures [21]. Daily nutrient intakes for each participant were computed using the United States Department of Agriculture’s (USDA) national nutrient databank [22]. Then the frequencies of consumed foods were transformed into daily intakes. The nutrient composition of all foods was derived by using modified nutritionist IV software [23]. To analyze dietary pattern, food items were grouped into 36 different categories. Food items were included in a certain food group based on the similarity of nutrients and their association with NAFLD. Definitions of food groups in terms of food frequency questionnaire items is presented in Table 1.

**Table 1:** Food groups used in the dietary pattern analysis

Food groups	Food items
Processed meats	Sausage, kielbasa
Red meats	Lamb, beef, ground meat
Organ meats	Lamb-tongue, (beef) lamb-brain, (beef) lamb tripe, meat of sheep's head, leg, lamb, liver, heart and kidney. (beef)
Fish	Fish, canned fish
Skinless poultry	Chicken no skin
Poultry-skin	Chicken with skin
Eggs	Egg
Low fat dairy	Low fat milk, without fat milk, yogurt, dried whey, cheese
High fat dairy	Milk high fat, cocoa milk, chocolate milk, Yogurt-plain-whole milk, cream, ice cream cheese-cream
Yogurt drink	Dough
Soft drink	Soft drink
Tea	Tea
Coffee	Coffee
Fruits	Cantaloupe, melon, watermelon, pear, apricot, cherry, apple, peach, nectarine, plum, fig, grape, kiwi, grapefruit, orange, persimmon, tangerine, pomegranate, date, black cherry, strawberry, banana, sweet lemon, lemon cranberry, pineapple, raisin, mulberry
Natural juices	Grapefruit juice, orange juice, apple juice, melonjuice
Canned fruits	Canned fruits, canned pineapple
Dried fruits	Dried fig, dried peach, dried apricot, dried mulberry
Vegetables	Lettuce, tomato, cucumber, fines herbs, pot-herb, Pumpkin, squash, eggplant, celery, pea, string bean, raw carrot, cookedcarrot, raw onion, fried onion, cabbage, cauliflower, sweet peppers, rawspinach, cooked spinach, turnip, cooked mushrooms, maize
Nuts	Seeds, walnuts, pistachios, hazelnuts, almonds. (pumpkin, sunflower, watermelon)
Legumes	Lentils, beans, chickpea, broad bean, soya, bean-mung, Pea
Whole grains	Dark breads (bavaria, sangak, tafton), cookedbarley, oat
Refined grains	White breads (lavash, baguettes), toast, rice, Spaghetti, vermicelli, pasta, wheat flour, biscuits
Fast foods	Fried potato, hamburger, pizza
Mayonnaise	Mayonnaise
Tomato sauce	Tomato sauce
Salty snacks	Crackers, cheese puffs, chips
Olive	Olive seed, olive oil
Sugar-sweets-desserts	Cake, sugar, honey, Jam, sugar, sweets, gaz (an Iranian confectionery made of sugar, nuts, andtamarisk), candy, sohan (an Iranian confectionery), chocolate, caramel, halva (kind of sweetmeat), donut
Hydrogenated fats	Solid oil, fat animal, animal oil, butter, margarine
Vegetable oils	(Except for olive oil) vegetable oil
Potato	Cooked potato
Garlic	Garlic
Condiments	Juice lime, black pepper
Salt	Salt
Pickles	Pickles
Broth	Broth

### Anthropometric assessment

Weight was measured using a standard digital Seca scale (made in Germany), while participants were unshod, wore minimum clothes and recorded to the nearest 100 g. Height was measured using a mounted tape in a standing relaxed shoulder position, unshod, to the nearest 0.5 cm. Body mass index (BMI) was calculated as weight (kg) divided by height in square meters (m^2^). Waist circumference (WC) and hip circumference were measured with a non-stretch tape applied horizontally in standing position over the light clothes. Waist was positioned as the narrowest circumference between the lower rib margin and the superior anterior iliac spine, and hip as the maximum level between the waist and thigh. All measurements were recorded to the nearest 0.5 cm. Waist to hip ratio (WHR) was calculated as waist circumference (cm) divided by hip circumference (cm). A trained dietitian conducted all the measurements in order to reduce error.

### Assessment of other variables

All participants were asked to answer demographic and socioeconomic questions including age, educational status, job, smoking status, home status, home type, foreign travel, income, and disease history. Many factors, including income, profession, housing, and education can determine socioeconomic status (SES) [24]. SES was defined based on educational level (academic and non-academic education), family size (≤ 4, > 4 people), acquisition (house ownership or not), and foreign travel (yes or no). For each variable of the SES score, participants were given a score of 1 if they had ≤ 4 family members, were academically educated, owned a house, or travelled internationally. Instead, they were given a score of 0 if they had > 4 family members, had non-academic education, had leasehold house or had not foreign travel. Then, total SES score was calculated by summing up the assigned scores (minimum SES score of 0 to maximum score of 4). Individuals with the score of 4 were considered as having high SES.

Data on physical activity was obtained via a short form of validated international physical activity questionnaire (IPAQ), which was presented as metabolic equivalent-minutes per week (MET-min/week) [25]. All questionnaires were completed by a trained dietitian through a personal interview in a relaxed atmosphere.

### Statistical analysis

To identify the major dietary patterns, based on 36 food groups, a factor analysis was conducted. Principal component analysis was used, and the factors were rotated by an orthogonal transformation (varimax rotation). The natural interpretation of the factors in conjunction with eigenvalues > 1 and the scree plot distinguished whether a factor had to be retained. Dietary patterns were labeled on the basis of our interpretation of the data and of the previous research. Summing intakes of food groups weighted by their factor loadings computed the factor score for each pattern and each participant received a factor score for each identified pattern. Participants were categorized based on tertiles of dietary pattern scores. To compare general characteristics across tertiles, we used one-way analysis of variance (ANOVA) and chi-square tests where appropriate. Energy-adjusted intakes of foods and nutrients across tertiles of dietary patterns were examined using analysis of covariance.

Multivariable-adjusted odds ratio using logistic regression were computed for evaluating the association of dietary patterns and NAFLD in different models. In the crude model only, the impact of dietary patterns with NAFLD was computed. In model I the association was adjustment for age (continuous), gender (categorical), education (under diploma, diploma, bachelor, higher than bachelor), and marital status (married, single, widowed or divorced). Further adjustment was made for BMI, smoking (yes or no) and physical activity (MET-min/ wk) in model II, additional adjustment for SES in model III and further adjustments for energy intake (kcal/d) in model IV. In all analyses, the first tertile of dietary patterns’ score was considered as a reference. To assess the overall trend of odds ratios across increasing tertiles of dietary pattern scores, we treated the tertile categories as an ordinal variable in the analyses. Multivariable logistic regression models were used to obtain adjusted OR. Covariates were the same as above. The Mantel–Haenszel extension chi-square test was used to assess the overall trend of OR across increasing tertiles of dietary pattern scores. P < 0.05 was considered significant. All analyses were performed using SPSS software (version 21; SPSS Inc, Chicago, IL, USA).

## Result

### Identified major dietary patterns

Factor analysis revealed three major dietary patterns from 36 food groups. The first one was labeled as “western pattern” which reflected the intakes of foods such as processed meats, organ meats, high fat dairy, soft drinks, refined grains, fast foods, mayonnaise, salty snacks, sugar-sweets-desserts and hydrogenated fats. The second pattern, was labeled “healthy pattern”, which displayed relatively high consumption of fish, skinless poultry, low fat dairy, fresh fruits, natural juices, canned fruits, dried fruits, vegetables, nuts, olive and garlic. The third pattern labeled “traditional pattern” and showed relatively high consumption of red meat organ meats, skinless poultry, eggs, yogurt drink, tea, legumes, tomato sauce, sugar-sweets-desserts, potato, condiments, salt, pickles and broth. Altogether, these three dietary patterns explained 50% of variance in primary variable. The factor loadings for each dietary pattern are presented in Table 2.

**Table 2:** Factor-loading matrix for major dietary patterns

Food groups	Western diet	Healthy diet	Traditional diet
Processed meats	0.63		–
Red meats	–	–	0.44
Organ Meats	0.31	–	0.32
Fish	–	0.32	–
Skinless poultry	–	0.23	0.21
Poultry-skin	–	–	–
Eggs	–	–	0.57
Low fat dairy	–	0.35	–
High fat dairy	0.56	–	–
Yogurt drink	–	–	0.27
Soft drink	0.64	–	–
Tea	–	–	0.31
Coffee	–	–	–
Fruits	–	0.62	–
Natural juices	–	0.45	–
Canned fruits	–	0.34	–
Dried fruits	–	0.48	–
Vegetables	–	0.50	0.25
Nuts	–	0.48	–
Legumes	–	–	0.48
Whole grains	–	–	–
Refined grains	0.30	–	–
Fast foods	0.65	–	–
Mayonnaise	0.51	–	–
Tomato sauce	–	–	0.23
Salty snacks	0.47	–	–
Olive	–	0.45	–
Sugar-sweets-desserts	0.40	–	0.32
Hydrogenated fats	0.55	–	–
Vegetable oils	–	–	–
Potato	–	–	0.56
Garlic	–	0.31	–
Condiments	–	0.26	0.42
Salt	–	–	0.20
Pickles	–	0.21	0.32
Broth	–	–	0.36

Values ≤ 0.20 were excluded for simplicity

### General characteristics and dietary intakes of study participants

The socio-demographic and lifestyle characteristics of the 225 cases and 450 controls are presented in Table 3. There were no statistically significant differences between case and control groups in terms of age, gender, waist-circumference and WHR. However, other variables including weight, BMI, physical activity, SES and smoking in controls were significantly different than cases (*P* value < 0.05). In addition, cases had a significantly higher recorded energy intake than the controls.

**Table 3:** General Characteristics of subjects with and without nonalcoholic fatty liver

Variables	Controls (n = 450)	Cases (n = 225)	*P* value^†^
Age (year)	37.88 ± 8.92	38.63 ± 8.71	0.72
Weight (kg)	65.59 ± 8.88	83.30 ± 10.61	0.03
BMI (kg^2^/m)	24.99 ± 3.09	30.56 ± 4.02	0.002
Waist-circumference (cm)	84.95 ± 12.05	102.28 ± 11.68	0.36
Hip-circumference (cm)	96.75 ± 6.03	104.95 ± 8.70	< 0.001
WHR	0.87 ± 0.06	0.97 ± 0.06	0.48
Physical activity (MET-min/wk)	1590.30 ± 949.44	1119.03 ± 616.35	< 0.001
SES	1.42 ± 0.78	1.96 ± 0.68	< 0.001
Energy (kcal)	2124 ± 187	2385 ± 164	0.004
Marital status			
Married	366 (81.3%)	199 (88.4%)	0.02
Married-p	14 (3.1%)	8 (3.6%)	
Single	70 (15.6%)	18 (8.0%)	
Education			
Lower than diploma	49 (10.9%)	33 (14.7%)	< 0.001
Diploma	186 (41.3%)	91 (40.4%)	
BSc	143 (31.8%)	94 (41.8%)	
Higher than BSc	72 (16%)	7 (3.1%)	
Home status			
Owner	323 (71.8%)	139 (61.8%)	
Tenant	124 (28.2%)	86 (38.2%)	
Foreign travel			
Yes	54 (12%)	53 (23.6%)	
No	396 (88%)	172 (76.4%)	
Family size			
> 4	135 (29.8%)	172 (68.8%)	
≤ 4	312 (70.2%)	78 (32.2%)	
Gender			
Male	233 (51.8%)	125 (55.6%)	
Female	217 (48.2%)	100 (44.4%)	
Smoking			
No	438 (97.3%)	209 (92.9%)	0.006
Yes	12 (2.7%)	16 (7.1%)	
Energy (kcal)	2124 ± 187	2385 ± 164	0.004

All values are mean ± SD

^†^Independent t-test was used for continuous variables and Chi-squared test for categorical variables

The comparison of demographic and lifestyle factors of study participants in different levels of major dietary pattern scores are summarized in Table 4. Distribution of participants in terms of gender, weight, physical activity, waist and hip circumference, WHR, BMI, SES, marital status and education were significantly different across western dietary pattern categories. Moreover, there were significant differences in WHR, age and marital status in the healthy dietary pattern categories. Furthermore, across different levels of traditional dietary pattern, there were no significantly different according to participant’s main characteristics.

**Table 4:** The comparison of demographic and lifestyle factors of study participants in different levels of major dietary patterns

Variable	Western dietary pattern				Healthy dietary pattern				Traditional dietary pattern			
	1	2	3	*P*^**†**^	1	2	3	*P*^**†**^	1	2	3	*P*^**†**^
Age (year)	38.69 ± 9.36	37.70 ± 8.43	37.72 ± 8.83	0.45	36.85 ± 8.61	38.35 ± 9.31	38.91 ± 8.60	0.04	37.08 ± 9.03	38.70 ± 8.95	38.32 ± 8.60	0.17
Weight (Kg)	65.48 ± 9.02	69.15 ± 12.60	78.63 ± 12.12	< 0.001	72.56 ± 12.53	70.22 ± 12.32	70.51 ± 12.95	0.13	70.64 ± 12.81	70.59 ± 12.52	72.05 ± 12.55	0.43
BMI (Kg/m^2^)	25.34 ± 3.17	26.21 ± 4.56	28.70 ± 4.27	< 0.001	26.97 ± 4.20	26.34 ± 4.01	26.94 ± 4.60	0.26	26.62 ± 4.12	26.64 ± 4.07	26.99 ± 4.64	0.64
Waist-circumference (cm)	85.62 ± 7.70	88.98 ± 11.04	96.79 ± 10.42	< 0.001	91.65 ± 10.92	89.96 ± 10.44	89.81 ± 11.20	0.17	90.24 ± 10.98	90.13 ± 10.42	91.05 ± 11.23	0.66
Hip-circumference (cm)	97.16 ± 6.50	98.95 ± 7.89	102.12 ± 8.40	< 0.001	99.72 ± 7.53	98.52 ± 7.77	100.01 ± 8.33	0.14	99.16 ± 7.35	98.95 ± 7.55	100.13 ± 8.71	0.28
WHR	0.88 ± 0.06	0.89 ± 0.06	0.94 ± 0.08	< 0.001	0.91 ± 0.08	0.91 ± 0.07	0.89 ± 0.07	0.01	0.90 ± 0.08	0.91 ± 0.07	0.90 ± 0.07	0.97
Physical activity (MET-min/wk)	1552 ± 892	1581 ± 973	1227 ± 776	< 0.001	1388 ± 800	1576 ± 938	1396 ± 938	0.06	1418 ± 890	1506 ± 976	1436 ± 820	0.59
SES	1.49 ± 0.80	1.48 ± 0.77	1.76 ± 0.73	< 0.001	1.59 ± 0.75	1.53 ± .77	1.61 ± .81	0.56	1.64 ± 0.78	1.48 ± 0.79	1.61 ± 0.76	0.09
Marital status												
Married	158 (81.0)	161 (82.1)	171 (87.2)	0.04	164 (84.1)	158 (80.6)	168 (85.7)	0.02	154 (79.0)	170 (86.7)	166 (84.7)	0.09
Married-p	10 (5.1)	2 (1.0)	4 (2.0)		1 (0.5)	11 (5.6)	4 (2)		4 (2.1)	7 (3.6)	5 (2.6)	
Single	27 (13.8)	33 (16.8)	21 (10.7)		30 (15.4)	27 (13.8)	24 (12.2)		37 (19)	19 (9.7)	25 (12.8)	
Education												
Lower than diploma	20 (10.3)	19 (9.7)	27 (13.8)	0.01	15 (7.7)	25 (12.8)	26 (13.3)	0.11	17 (8.7)	31 (15.8)	18 (9.2)	0.22
Diploma	81 (41.5)	83 (42.3)	75 (38.3)		76 (39)	75 (38.3)	88 (44.9)		83 (42.6)	81 (41.3)	75 (38.3)	
BSc	65 (33.3)	63 (32.1)	83 (42.3)		80 (41)	66 (33.7)	65 (33.2)		70 (35.9)	62 (31.6)	79 (40.3)	
Higher than BSc	29 (14.9)	31 (15.8)	11 (5.6)		24 (12.3)	30 (15.3)	17 (8.7)		25 (12.8)	22 (11.2)	24 (12.2)	
NAFLD												
YES	6 (3.1)	40 (20.4)	142 (72.4)	< 0.001	75 (38.5)	60 (30.6)	53 (27)	0.06	66 (33.8)	56 (28.6)	66 (33.7)	0.44
Gender												
Male	90 (46.2)	103 (52.6)	118 (60.2)	0.02	106 (54.4)	112 (57.1)	93 (47.4)	0.14	98 (50.3)	108 (55.1)	105 (53.6)	0.61
Female	105 (53.8)	93 (47.4)	78 (39.8)		89 (45.6)	84 (42.9)	103 (52.6)		97 (49.7)	88 (44.9)	91 (46.4)	

All values are mean ± SD

^†^ANOVA for continuous variables and Chi-squared test for categorical variable

The comparison of macro and micro-nutrients intake in different categories of major dietary patterns is illustrated in Table 5. Participants in all three tertiles of western dietary pattern had significantly different intakes of energy, protein, carbohydrate, saturated fat, linoleic fat, oleic fat, total sugar, sucrose, lactose, copper, zinc, vitamin E, vitamin C. Furthermore, comparison of nutrient intakes in the healthy dietary pattern categories showed significant differences in terms of energy, protein, cholesterol, linoleic fat, EPA, DHA, total dietary fiber, Total sugar, glucose, fructose, lactose, galactose, Cu, Zn, vitamin A, vitamin E, and vitamin C intakes. Moreover, participants in the different tertile of traditional dietary pattern had significantly different intakes of energy, protein, carbohydrate, cholesterol, oleic fat, total sugar, glucose, fructose, lactose, galactose, Fe, Zn, vitamin E, vitamin C.

**Table 5:** The comparison of macro and micro-nutrients intakes in different categories of major dietary patterns

Nutrients*	Western dietary pattern				Healthy dietary pattern				Traditional dietary pattern			
	1	2	3	*P*	1	2	3	*P*	1	2	3	*P*
Protein (gr)	74.47 ± 0.90	73.01 ± 0.87	69.09 ± 0.90	< 0.001	68.39 ± 0.88	73.03 ± 0.86	75.12 ± 0.89	< 0.001	68.32 ± 0.91	72.47 ± 0.86	75.75 ± 0.92	< 0.001
Carbohydrate (gr)	306.50 ± 2.87	310.55 ± 2.77	300.17 ± 2.86	0.03	306.15 ± 2.85	302.33 ± 2.79	308.74 ± 2.87	0.27	314.80 ± 2.92	306.07 ± 2.75	296.39 ± 2.95	< 0.001
Fat (gr)	74.57 ± 1.23	72.60 ± 1.19	78.06 ± 1.23	0.06	74.58 ± 1.23	75.95 ± 1.20	74.69 ± 1.24	0.67	73.30 ± 1.27	74.57 ± 1.20	77.35 ± 1.28	0.09
Saturated fat (gr)	25.02 ± 0.54	24.46 ± 0.53	26.77 ± 0.54	< 0.001	24.52 ± 0.54	25.98 ± 0.53	25.74 ± 0.54	0.12	24.86 ± 0.56	25.30 ± 0.53	25.10 ± 0.57	0.64
Cholesterol(gr)	212.49 ± 8.47	221.70 ± 8.17	214.06 ± 8.44	0.69	209.92 ± 8.37	221.85 ± 8.19	216.47 ± 8.43	< 0.001	178.45 ± 8.37	209.22 ± 7.91	260.41 ± 8.47	< 0.001
Linoleic fat (gr)	13.33 ± 0.37	12.99 ± 0.36	14.23 ± 0.37	0.04	14.51 ± 0.37	13.49 ± 0.36	12.54 ± 0.37	< 0.001	12.90 ± 0.38	13.38 ± 0.36	14.26 ± 0.39	0.06
Oleic fat (gr)	23.61 ± 0.48	22.68 ± 0.46	24.64 ± 0.48	0.01	24.15 ± 0.48	23.82 ± 0.47	22.96 ± 0.48	0.21	22.71 ± 0.49	23.27 ± 0.46	24.93 ± 0.50	0.008
EPA (gr)	0.022 ± 0.002	0.023 ± 0.002	0.021 ± 0.002	0.74	0.01 ± 0.002	0.02 ± 0.002	0.03 ± 0.002	< 0.001	0.02 ± 0.002	0.024 ± 0.002	0.021 ± 0.002	0.24
DHA (gr)	0.07 ± 0.006	0.08 ± 0.006	0.07 ± 0.006	0.65	0.05 ± 0.006	0.08 ± 0.006	0.10 ± 0.006	< 0.001	0.07 ± 0.006	0.08 ± 0.006	0.07 ± 0.006	0.21
Total dietary Fiber (gr)	38.32 ± 1.24	35.27 ± 1.19	34.33 ± 1.23	0.07	38.85 ± 1.22	34.54 ± 1.19	34.54 ± 1.23	0.01	37.09 ± 1.27	35.61 ± 1.20	35.22 ± 1.29	0.57
Total sugar (gr)	115.48 ± 2.14	112.23 ± 2.06	102.90 ± 2.13	< 0.001	93.40 ± 1.91	109.96 ± 1.87	127.15 ± 1.93	< 0.001	114.23 ± 2.21	110.56 ± 2.09	105.81 ± 2.23	0.04
Glucose (gr)	14.82 ± 0.42	14.67 ± 0.41	13.68 ± 0.42	0.13	10.61 ± 0.35	13.87 ± 0.34	18.67 ± 0.35	< 0.001	15.90 ± 0.43	13.77 ± 0.40	13.50 ± 0.43	< 0.001
Fructose (gr)	17.33 ± 0.50	16.91 ± 0.49	15.86 ± 0.50	0.12	12.15 ± 0.42	16.23 ± 0.41	21.69 ± 0.42	< 0.001	18.66 ± 0.51	15.96 ± 0.48	15.49 ± 0.52	< 0.001
Sucrose (gr)	30.75 ± 1.31	32.02 ± 1.27	27.13 ± 1.31	0.02	28.33 ± 1.30	29.63 ± 1.27	31.93 ± 1.31	0.16	28.16 ± 1.35	30.37 ± 1.27	31.36 ± 1.36	0.26
Lactose (gr)	18.55 ± 0.72	14.51 ± 0.70	14.26 ± 0.72	< 0.001	11.77 ± 0.70	17.02 ± 0.69	18.50 ± 0.71	< 0.001	16.86 ± 0.75	16.49 ± 0.71	13.95 ± 0.76	0.01
Galactose (gr)	3.50 ± 0.16	3.19 ± 0.15	3.18 ± 0.16	0.29	2.59 ± 0.15	3.56 ± 0.15	3.71 ± 0.15	< 0.001	3.34 ± 0.16	3.56 ± 0.15	2.97 ± 0.16	0.03
Fe (mg)	27.01 ± 0.93	25.83 ± 0.90	24.58 ± 0.93	0.20	25.94 ± 0.92	26.11 ± 0.90	25.36 ± 0.93	0.83	22.97 ± 0.94	25.42 ± 0.89	29.00 ± 0.95	< 0.001
Cu (mg)	1.42 ± 0.02	1.43 ± 0.02	1.36 ± 0.02	0.04	1.37 ± 0.02	1.36 ± 0.02	1.47 ± 0.02	< 0.001	1.40 ± 0.02	1.38 ± 0.02	1.43 ± 0.02	0.37
Zn (mg)	10.85 ± 0.13	10.65 ± 0.13	10.21 ± 0.13	< 0.001	10.00 ± 0.13	10.73 ± 0.13	10.98 ± 0.13	< 0.001	10.10 ± 0.14	10.64 ± 0.13	10.96 ± 0.14	< 0.001
Vit. A (RAE)	485.85 ± 19.79	459.96 ± 19.10	428.78 ± 19.73	0.14	337.24 ± 18.13	438.15 ± 17.74	598.45 ± 18.26	< 0.001	450.88 ± 20.27	435.74 ± 19.15	487.79 ± 20.50	0.18
Vit.E (mg)	11.39 ± 0.25	10.54 ± 0.24	10.75 ± 0.25	0.04	10.41 ± 0.24	10.98 ± 0.24	11.29 ± 0.25	0.04	10.59 ± 0.25	10.58 ± 0.24	11.51 ± 0.26	0.02
Vit.C (mg)	139.84 ± 5.09	129.47 ± 4.91	120.22 ± 5.07	0.03	83.52 ± 4.18	122.97 ± 4.09	182.76 ± 4.21	< 0.001	139.21 ± 5.22	123.04 ± 4.93	127.28 ± 5.28	0.07
Energy (kcal)ª	1906.38 ± 38.67	2135.52 ± 38.43	2382.59 ± 38.56	< 0.001	1954.34 ± 39.06	2091.77 ± 38.89	2358.63 ± 38.98	< 0.001	1839.06 ± 36.27	2091.40 ± 36.14	2473.69 ± 36.10	< 0.001

All values are presented as mean ± SD; the comparison of macro and micro-nutrients intakes were adjusted with the variables of age, gender, and energy and energy were adjusted with age and gender by the covariance analysis method (ANCOVA)

### The relationship between major dietary patterns and NAFLD

Crude and Multivariable-adjusted odds ratios and their 95% confidence interval of the associations between dietary patterns and NAFLD are presented in Table 6. In all models, tertile 1 (the lowest categories of adherence to dietary patterns) was defined as the reference category. Participants in the highest tertile of adherence to the western dietary pattern had significantly 2.04 times more chance for being NAFLD than those in the lowest tertile in the crude model (OR: 2.04; 95% CI: 1.13–3.92). After adjusting for the potential confounding factors, there was a positive significant relationship between affected with NAFLD and adherence to the western dietary pattern in the model I (OR: 2.61; 95% CI: 1.41–4.28), model II (OR: 2.82; 95% CI: 1.68–4.80), model III (OR: 3.11; 95% CI: 2.41–5.12) and in the model IV (OR: 3.64; 95% CI: 2.52–5.32). Furthermore, individuals in the highest tertile of adherence to the healthy dietary pattern were 41% less likely to have NAFLD, compared with those in the bottom category in the crude model (OR: 0.59; 95% CI: 0.38–0.90). The healthy dietary pattern was associated with a pronounced decreased risk of NAFLD, even after adjusting for potential confounding factors in model I (OR: 0.54; 95% CI: 0.34–0.83), model II (OR: 0.42; 95% CI: 0.23–0.78), model III (OR: 0.31; 95% CI: 0.18–0.54), model IV (OR: 0.30; 95% CI: 0.13–0.68). This study observed a protective role for traditional dietary pattern against on NAFLD in crude model (OR:0.50; 95% CI:0.37–0.68); however, this association was not significant after adjusting for the potential confounding variables in model I (OR: 0.98; 95% CI: 0.64–1.49), model II (OR: 0.75; 95% CI: 0.45–1.26), model III (OR: 0.74; 95% CI: 0.44–1.26), model IV (OR: 1.21; 95% CI: 0.57–2.57).

**Table 6:** Crude and multivariable-adjusted odds ratios and their 95% confidence interval of the associations between NAFLD and dietary patterns

	Tertile of Western dietary pattern				Tertile of Healthy dietary pattern				Tertile of Traditional dietary pattern			
	1	2	3	P-trend	1	2	3	P-trend	1	2	3	P-trend
Crude model	1	1.94 (1.08–3.82)	2.04 (1.13–3.92)	< 0.001	1	0.70 (0.46–1.07)	0.59 (0.38–0.90)	0.04	1	0.40 (0.29–0.54)	0.50 (0.37–0.68)	< 0.001
Model I^a^	1	2.24 (1.62–4.12)	2.61 (1.41–4.28)	< 0.001	1	0.66 (0.43–1.02)	0.54 (0.34–0.83)	0.01	1	0.72 (0.47–1.10)	0.98 (0.64–1.49)	0.26
Model II^b^	1	2.08 (1.14–3.78)	2.82 (1.46–4.80)	< 0.001	1	0.65 (0.36–1.20)	0.42 (0.23–0.78)	0.02	1	0.45 (0.26–0.76)	0.75 (0.45–1.26)	0.01
Model III^c^	1	2.72 (1.48–4.59)	3.11 (2.41–5.12)	< 0.001	1	0.53 (0.31–0.91)	0.31 (0.18–0.54)	< 0.001	1	0.49 (0.29–0.83)	0.74 (0.44–1.26)	0.03
Model IV^d^	1	3.17 (2.35–5.61)	3.64 (2.52–5.32)	< 0.001	1	0.52 (0.28–0.96)	0.30 (0.13–0.68)	0.01	1	0.57 (0.31–1.04)	1.21 (0.57–2.57)	0.03

^a^_Model I: adjusted for age, gender, education and marital status_

^b^_Model II: adjusted for age, gender, education, marital status, BMI, smoking and physical activity_

^c^_Model III: adjusted for age, gender, education, marital status, BMI, smoking, physical activity and SES_

^d^_Model IV: adjusted for age, gender, education, marital status, BMI, smoking, physical activity, SES, energy_

## Discussion

The current study investigated the association of three dietary patterns of "western dietary pattern", “healthy dietary pattern”, and "traditional dietary pattern” in Iranian adults with NAFLD. The "western dietary pattern", which mainly consists of a high intake of fast foods, soft drinks, processed meat, high-fat dairy products, hydrogenated fats, mayonnaise, salty snacks, sugar-sweet desserts, organ meats, and refined grains was significantly associated with the risk of NAFLD. This association was independent of age, gender, BMI, smoking, physical activities, SES, and energy intake. This finding indicated the negative role of diet which is enriched by fats and sweeteners. A previous study by Ritchiev et al. [26] also supported the results of the current study. The participants in the highest tertile of the "western dietary pattern" had the highest risk of NAFLD in comparison to the lowest tertile. In Zelber-Sagi et al. [27], it was demonstrated that high consumption of soft drinks was associated with the increasing risk of NAFLD in 375 adults. Moreover, it was explicated that high consumption of soft drinks increases the risk of NAFLD due to the high caloric content and/or the excessive amount of sugar (such as fructose) in these drinks. In addition, excessive fructose consumption is known to increase the risk of metabolic syndrome and its components, such as dyslipidemia, insulin resistance, and hypertension [14]. Moreover, refined grains, white bread, and sugar-sweets desserts, which are constituents of the "western dietary pattern", rapidly increase the insulin and glucose levels in blood, which cause insulin resistance, diabetes, and obesity [28]. Furthermore, rapid increase in blood sugar enhances the rate of “de-novo” synthesis and increases fat in liver cells [29]. It has been indicated both in animal [30] and human studies [31] that high glycemic index diet increases the fat accumulation in the liver cells and leads to hepatic steatosis. Moreover, "western dietary pattern" contains high amounts of saturated and trans fatty acids, which may affect the hepatic cells steatosis via chylomicron uptake after consuming fatty foods.

The participants in the highest tertile of “Healthy dietary pattern” had the lowest risk of affecting to NAFLD compared to the ones in the lowest tertile. “Healthy dietary pattern” is defined by high consumption of fruits, vegetables, nuts, olive oil, low-fat dairy products, fish and garlic. The current study found an inverse relation between the “healthy dietary pattern” and the risk of NAFLD, which was independent of age, gender, BMI, physical activities, SES, and energy intake. This effect could be as a consequence of high consumption of fruits and vegetables, which increases the intake of antioxidant vitamins, such as vitamins A, E, and C. Studies have shown that consuming antioxidant vitamins has a protective role against oxidative stress [32], and the risk of NAFLD [33]. Moreover, fruits and vegetables in the “Healthy dietary pattern” represent good sources of dietary fibers, which have an inverse association with insulin resistance and, thus, may conceivably reduce the risk of NAFLD [33]. Fish have high amounts of poly unsaturated fatty acids (Omega 3) which are capable of reducing total cholesterol and has a protective role against NAFLD [34, 35]. Similar to the “Healthy dietary pattern”, a protective effect of the Mediterranean diet, which is defined as a diet rich in olive oil, fresh fruits, nuts, and vegetables; moderate in dairy products, fish, poultry and red wine; and low in red meat, eggs, sweets and processed foods [36], has been shown previously by prospective [37] and intervention studies [38, 39].

The traditional dietary pattern is different depending on the region or country, and encompasses the common foods in that country. In Esmaeillzadeh et al. [40], a traditional dietary pattern was represented by a high intake of broth, legumes, tea, whole grains and potato. In another study, traditional dietary pattern was characterized by high intake of potatoes, beans, red meat, eggs and dried fruits in men and women [41]. In the present study, “traditional dietary pattern” (due to the fact that food groups which are loaded in factor-loading analysis in third dietary pattern are similar to those of that in Iranian routine diet, we dubbed it traditional dietary pattern) consisted of high consumption of red meat organ meats, skinless poultry, eggs, yogurt drink, tea, legumes, tomato sauce, sugar-sweets-desserts, potato, condiments, salt, pickles and broth. The current study did not find any significant association between "traditional dietary pattern" and risk of NAFLD. The complex nature of this dietary pattern, which is formed by different dietary components, may have precluded significant associations. The “traditional dietary pattern” comprises healthy and unhealthy foods; and whilst healthy foods have a protective role against the emergence of NAFLD, and unhealthy foods increase the risk of NAFLD. In other words, this dietary pattern included several food items which have been reported to have negative impact on NAFLD risk factors such as red meat, organ meat, broth [42], sugar-sweets-desserts [43] and salt [44]; however, there are also food items with anti-inflammatory and anti-oxidative function. Curcumin, cinnamon, cardamom and ginger which are the most common condiments used by Iranian people have potentially liver protective effect [45–48]. In addition, tea is the habitual drink among Iranian people. It has been suggested that drinking tea could prevent incidence of NAFLD by its catching and polyphenol components [49]. In this regard, it is possible that the different effect of included food groups cause of non-significant result.

Our study has limitations that need to be taken into consideration. We distinguished dietary patterns by using food intake data only, while the inclusion of eating behaviors such as meal and snack patterns in the dietary pattern analysis should recommended in future studies. Restriction of the FFQ also applies to dietary pattern analyses that are based on dietary information accumulated by this method. Also, several steps in factor analysis, such as grouping of different food items, definition of a number of factors, and interpretation of those factors, were subjective. It should be noted that each pattern was also connected to other risk indicators such as BMI and socioeconomic status (SES), although we adjusted for these factors, we cannot prevent some residual confounding. In addition, due to the nature of observational studies, the causal relationships between dietary patterns and odds of NAFLD could not be made, and we could not clarify whether adherence to the diet modifies the risk of NAFLD or the disease could influence food preferences. Further studies by which the metabolic features associated to NAFLD are also considered, are necessary to confirm the vicinity of the findings.

## Conclusion

In conclusion, the present study indicated that a dietary pattern characterized by high intakes of fruits, vegetables, nuts, olive oil, low-fat dairy products, fish, and garlic was associated with reduced risk of NAFLD, while a dietary pattern with high amounts of fast foods, soft drinks, processed meat, high-fat dairy products, hydrogenated fats, mayonnaise, salty snacks, sugar-sweet desserts, organ meats, and refined grains was linked with a greater risk of NAFLD in Iranian adults. These findings should be adopted and operationalized by key stakeholders in the treatment and management of NAFLD.

## Data Availability

The datasets generated and analyzed during the current study are not publicly available, due to institutional policy, but may be requested directly from the corresponding author.

## Abbreviations

NAFLD: Non-alcoholic fatty liver disease
T2DM: Type 2 diabetes mellitus
FFQ: Food frequency questionnaire
USDA: United States Department of Agriculture
BMI: Body mass index
WC: Waist circumference
WHR: Waist to hip ratio
SES: Socioeconomic status
IPAQ: International physical activity questionnaire
OR: Odd ratio
EPA: Eicosapentaenoic acid
DHA: Docosahexaenoic
